# 1-Piperonylpiperazinium picrate

**DOI:** 10.1107/S1600536814001524

**Published:** 2014-01-29

**Authors:** Channappa N. Kavitha, Manpreet Kaur, Brian J. Anderson, Jerry P. Jasinski, H. S. Yathirajan

**Affiliations:** a Department of Studies in Chemistry, University of Mysore, Manasagangotri, Mysore 570 006, India; b Department of Chemistry, Keene State College, 229 Main Street, Keene, NH 03435-2001, USA

## Abstract

In the cation of the title salt [systematic name: 4-(2*H*-1,3-benzodioxol-5-ylmeth­yl)piperazin-1-ium 2,4,6-tri­nitro­phen­o­late], C_12_H_17_N_2_O_2_^+^·C_6_H_2_N_3_O_7_^−^, the piperazine ring adopts a slightly disordered chair conformation. The piperonyl ring system and the piperazine ring are twisted with respect to each other with an N—C—C—C torsion angle of 40.7 (2)°. In the anion, the dihedral angles between the mean planes of the nitro substituents *ortho* to the phenolate O atom and the mean plane of the phenyl ring are 28.8 (9) and 32.2 (8)°. In contrast, the nitro group in the *para* position lies much closer to the aromatic ring plane, subtending a dihedral angle of 3.0 (1)°. In the crystal, the cations and anions inter­act through N—H⋯O hydrogen bonds and a weak C—H⋯O inter­action. Weak C—H⋯O inter­actions are also observed between the anions, forming *R*_2_^2^(10) graph-set ring motifs. In addition, a weak centroid–centroid π–π stacking inter­action between the aromatic rings of the cation and the anion, with an inter­centroid distance of 3.7471 (9) Å, contributes to the crystal packing, resulting in a two-dimensional network along (10-1).

## Related literature   

For pharmaceutical applications of the title cation, see: Millan *et al.* (2001 ▶) and for the pharmacological and toxicological uses of piperazine derivatives, see: Brockunier *et al.* (2004 ▶); Bogatcheva *et al.* (2006 ▶); Choudhary *et al.* (2006 ▶); Elliott (2011 ▶); Kharb *et al.* (2012 ▶). For a related structure, see: Capuano *et al.* (2000 ▶). For puckering parameters, see: Cremer & Pople (1975 ▶) and for standard bond lengths, see: Allen *et al.* (1987 ▶).
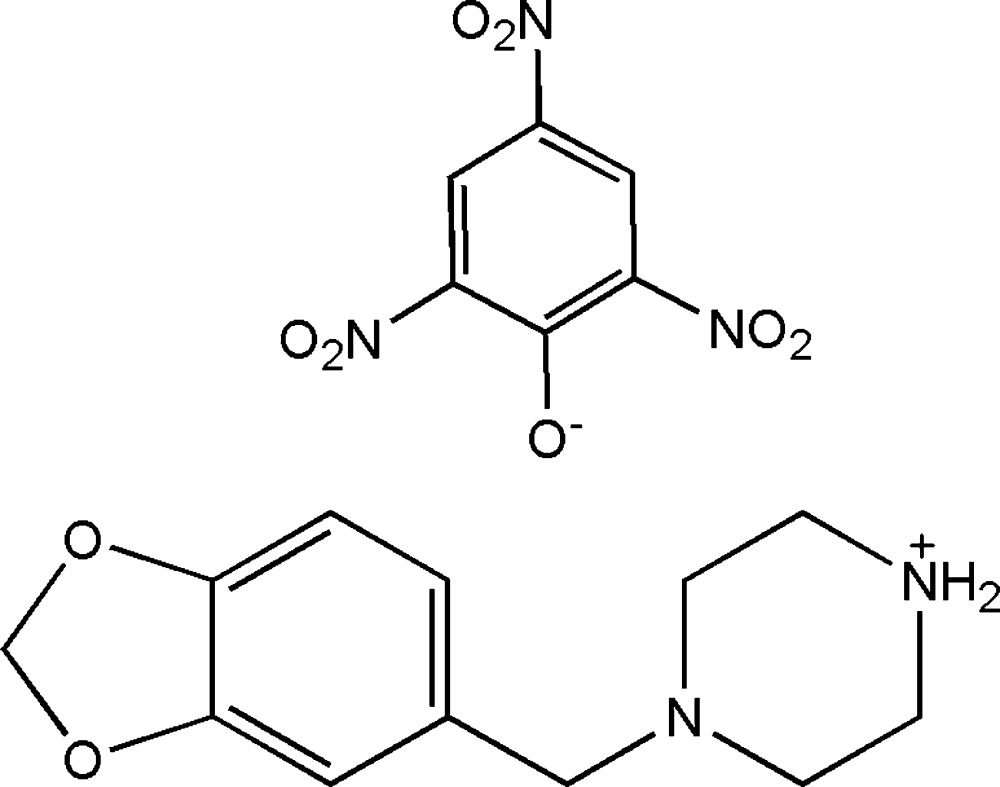


## Experimental   

### 

#### Crystal data  

C_12_H_17_N_2_O_2_^+^·C_6_H_2_N_3_O_7_^−^
*M* *_r_* = 449.38
Monoclinic, 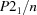

*a* = 12.0864 (2) Å
*b* = 6.96981 (11) Å
*c* = 23.4898 (4) Å
β = 96.5141 (17)°
*V* = 1965.99 (6) Å^3^
*Z* = 4
Cu *K*α radiation
μ = 1.06 mm^−1^
*T* = 173 K
0.48 × 0.24 × 0.22 mm

#### Data collection  

Agilent Gemini EOS diffractometer
Absorption correction: multi-scan (*CrysAlis PRO* and *CrysAlis RED*; Agilent, 2012 ▶) *T*_min_ = 0.761, *T*_max_ = 1.000
12154 measured reflections
3837 independent reflections
3316 reflections with *I* > 2σ(*I*)
*R*_int_ = 0.033

#### Refinement  

*R*[*F* ^2^ > 2σ(*F* ^2^)] = 0.043
*wR*(*F* ^2^) = 0.121
*S* = 1.05
3837 reflections
298 parameters
H atoms treated by a mixture of independent and constrained refinement
Δρ_max_ = 0.29 e Å^−3^
Δρ_min_ = −0.21 e Å^−3^

### 

Data collection: *CrysAlis PRO* (Agilent, 2012 ▶); cell refinement: *CrysAlis PRO*; data reduction: *CrysAlis RED* (Agilent, 2012 ▶); program(s) used to solve structure: *SUPERFLIP* (Palatinus & Chapuis, 2007 ▶); program(s) used to refine structure: *SHELXL2012* (Sheldrick, 2008 ▶); molecular graphics: *OLEX2* (Dolomanov *et al.*, 2009 ▶); software used to prepare material for publication: *OLEX2*.

## Supplementary Material

Crystal structure: contains datablock(s) I. DOI: 10.1107/S1600536814001524/sj5385sup1.cif

Structure factors: contains datablock(s) I. DOI: 10.1107/S1600536814001524/sj5385Isup2.hkl

Supporting information file. DOI: 10.1107/S1600536814001524/sj5385Isup3.cml

CCDC reference:

Additional supporting information:  crystallographic information; 3D view; checkCIF report

## Figures and Tables

**Table 1:** Hydrogen-bond geometry (Å, °)

*D*—H⋯*A*	*D*—H	H⋯*A*	*D*⋯*A*	*D*—H⋯*A*
N2*A*—H2*AA*⋯O1*B* ^i^	0.91 (2)	1.86 (3)	2.7409 (19)	163 (2)
N2*A*—H2*AB*⋯O1*B* ^ii^	0.91 (2)	1.91 (2)	2.7798 (18)	159 (2)
C4*A*—H4*A*⋯O6*B* ^iii^	0.95	2.48	3.335 (2)	150
C3*B*—H3*B*⋯O3*B* ^iv^	0.95	2.54	3.473 (2)	166

Symmetry codes: (i) 
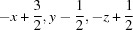
; (ii) 
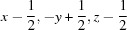
; (iii) 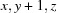; (iv) 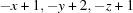.

**Fig. 1.**
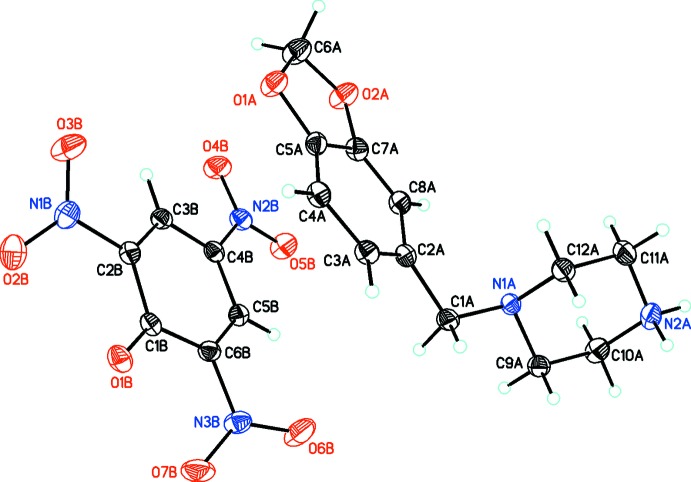
ORTEP drawing of (I) (C12H17N2O2+. C6H2N3O7-) showing the labeling scheme with 50% probability displacement ellipsoids.

**Fig. 2.**
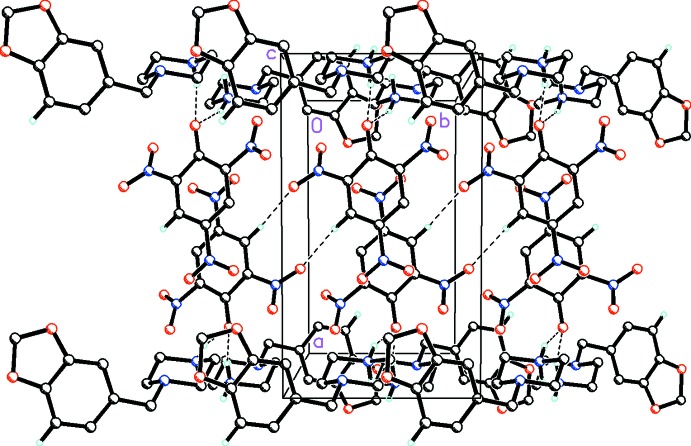
Molecular packing for (I) viewed along the c axis. Dashed lines indicate N—H···O intermolecular hydrogen bonds and weak C—H···O intermolecular interactions. H atoms not involved in hydrogen bonding have been removed for clarity.

## supplementary crystallographic information

### 1. Comment

1-(3,4-Methylenedioxybenzyl)piperazine or 1-piperonylpiperazine is a psychoactive drug of the piperazine class and is used to synthesise the drug, piribedil, an antiparkinsonian agent (Millan *et al.*, 2001). The piperazine moiety is extensively employed to construct various bioactive molecules with anti-bacterial or antimalarial activity and as antipsychotic agents (Choudhary *et al.*, 2006). A valuable insight into recent advances on antimicrobial activity of piperazine derivatives is provided by Kharb *et al.*, (2012). Piperazines are among the most important building blocks in today's drug discovery and are found in biologically active compounds across a number of different therapeutic areas (Brockunier *et al.*, 2004; Bogatcheva *et al.* , 2006). A review on the current pharmacological and toxicological information for piperazine derivatives is available (Elliott, 2011). The crystal structure of an N-piperonyl analogue of the atypical antipsychotic clozapine (Capuano *et al.*, 2000) has been reported. In view of the above importance of piperonylpiperazines, this paper reports the crystal structure of the title salt, (I), C_12_H_17_N_2_O_2_^+^. C_6_H_2_N_3_O_7_^-^.

The asymmetric unit of the title salt, (I), C_12_H_17_N_2_O_2_^+^ . C_6_H_2_N_3_O_7_^-^, consists of a monoprotonated 1-piperonylpiperazinium cation and a picrate anion (Fig.1). In the cation, the piperazine ring adopts a slightly disordered chair conformation (puckering parameters Q, θ, and φ = 0.5877 (18)Å, 2.28 (16) ° and 6(5) °; (Cremer & Pople, 1975). The piperonyl ring system and the piperazine rings are twisted with respect to each other with an N1A/C1A/C2A/C8A torsion angle of 40.7 (2)°. In the anion, the dihedral angles between the mean planes of the nitro substituents ortho to the phenolate O atom and the mean plane of the phenyl ring are 28.8 (9)° (C6B/N3B/O7B/O6B) and 32.2 (8)°(N1B/O3B/O2B/C2B), respectively. In contrast, the nitro group in the para position lies much closer to the aromatic ring plane, subtending dihedral angles of 3.0 (1)°. Bond lengths are in normal ranges (Allen *et al.*, 1987). In the crystal, the cations and anions interact through intermolecular N—H···O hydrogen bonds and a weak C3B—H3B···O3B intermolecular interaction (Fig.2). Weak C—H···O intermolecular interactions are also observed between adjacent anions forming R_2_^2^(10) graph set ring motifs. In addition, a weak Cg3–Cg5 π–π stacking interaction with an intercentroid distance of 3.7471 (9)Å (symmetry operation x, y, z; Cg3 and Cg5 are the centroids of the C2A–C8A and C1B–C6B rings respectively) contribute to the crystal packing resulting in a 2D network along (1 0 -1).

### 2. Experimental

1-piperonylpiperazine ( 2.2g, 0.01 mol) and picric acid (2.29 g, 0.01 mol), were dissolved in hot N,N-dimethylformamide and stirred for 10 mins at 323 K. The resulting solution was allowed to cool slowly at room temperature. Crystals of the title salt appeared after a few days (m. p: 463-468K).

### 3. Refinement

H2AA and H2AB were located in a difference map and refined isotropically. All of the other H atoms were placed in calculated positions and refined using the riding model with C—H bond lengths of 0.95Å (CH) or 0.99Å (CH_2_). Isotropic displacement parameters for these atoms were set to 1.2 (CH, CH_2_) times *U*_eq_ of the parent atom.

### Crystal data

**Table:**

C_12_H_17_N_2_O_2_^+^·C_6_H_2_N_3_O_7_^−^	*F*(000) = 936
*M**_r_* = 449.38	*D*_x_ = 1.518 Mg m^−^^3^
Monoclinic, *P*2_1_/*n*	Cu *K*α radiation, λ = 1.5418 Å
*a* = 12.0864 (2) Å	Cell parameters from 4971 reflections
*b* = 6.96981 (11) Å	θ = 3.7–72.4°
*c* = 23.4898 (4) Å	µ = 1.06 mm^−^^1^
β = 96.5141 (17)°	*T* = 173 K
*V* = 1965.99 (6) Å^3^	Irregular, dark yellow
*Z* = 4	0.48 × 0.24 × 0.22 mm

### Data collection

**Table:**

Agilent Gemini EOS diffractometer	3837 independent reflections
Radiation source: Enhance (Cu) X-ray Source	3316 reflections with *I* > 2σ(*I*)
Detector resolution: 16.0416 pixels mm^-1^	*R*_int_ = 0.033
ω scans	θ_max_ = 72.5°, θ_min_ = 3.8°
Absorption correction: multi-scan (*CrysAlis PRO* and *CrysAlis RED*; Agilent, 2012)	*h* = −13→14
*T*_min_ = 0.761, *T*_max_ = 1.000	*k* = −6→8
12154 measured reflections	*l* = −28→28

### Refinement

**Table:**

Refinement on *F*^2^	Hydrogen site location: mixed
Least-squares matrix: full	H atoms treated by a mixture of independent and constrained refinement
*R*[*F*^2^ > 2σ(*F*^2^)] = 0.043	*w* = 1/[σ^2^(*F*_o_^2^) + (0.0679*P*)^2^ + 0.5476*P*] where *P* = (*F*_o_^2^ + 2*F*_c_^2^)/3
*wR*(*F*^2^) = 0.121	(Δ/σ)_max_ < 0.001
*S* = 1.05	Δρ_max_ = 0.29 e Å^−^^3^
3837 reflections	Δρ_min_ = −0.21 e Å^−^^3^
298 parameters	Extinction correction: *SHELXL*, Fc^*^=kFc[1+0.001xFc^2^λ^3^/sin(2θ)]^-1/4^
0 restraints	Extinction coefficient: 0.0016 (2)
Primary atom site location: structure-invariant direct methods	

### Special details

**Table:**

Geometry. All esds (except the esd in the dihedral angle between two l.s. planes) are estimated using the full covariance matrix. The cell esds are taken into account individually in the estimation of esds in distances, angles and torsion angles; correlations between esds in cell parameters are only used when they are defined by crystal symmetry. An approximate (isotropic) treatment of cell esds is used for estimating esds involving l.s. planes.

### Fractional atomic coordinates and isotropic or equivalent isotropic displacement parameters (Å^2^)

**Table:**

	*x*	*y*	*z*	*U*_iso_*/*U*_eq_	
O1B	0.84597 (9)	0.58513 (17)	0.47124 (5)	0.0352 (3)	
O2B	0.77598 (13)	0.8599 (2)	0.53866 (6)	0.0578 (4)	
O3B	0.64101 (13)	1.01645 (19)	0.49224 (7)	0.0570 (4)	
O4B	0.32910 (10)	0.61289 (19)	0.42367 (6)	0.0463 (3)	
O5B	0.35919 (10)	0.34651 (18)	0.38117 (6)	0.0461 (3)	
O6B	0.73947 (12)	0.1561 (2)	0.36561 (7)	0.0598 (4)	
O7B	0.85686 (11)	0.2174 (2)	0.43946 (7)	0.0571 (4)	
N1B	0.69547 (12)	0.8710 (2)	0.50202 (6)	0.0372 (3)	
N2B	0.39201 (11)	0.4894 (2)	0.40823 (6)	0.0331 (3)	
N3B	0.76868 (11)	0.2469 (2)	0.40934 (6)	0.0372 (3)	
C1B	0.74326 (12)	0.5605 (2)	0.45716 (6)	0.0274 (3)	
C2B	0.66022 (13)	0.6984 (2)	0.46929 (6)	0.0285 (3)	
C3B	0.54835 (13)	0.6790 (2)	0.45310 (6)	0.0286 (3)	
H3B	0.4975	0.7755	0.4621	0.034*	
C4B	0.51081 (12)	0.5151 (2)	0.42331 (6)	0.0277 (3)	
C5B	0.58320 (13)	0.3750 (2)	0.40867 (6)	0.0283 (3)	
H5B	0.5563	0.2651	0.3875	0.034*	
C6B	0.69516 (12)	0.3982 (2)	0.42543 (6)	0.0288 (3)	
O1A	0.46181 (10)	0.97115 (18)	0.33755 (6)	0.0418 (3)	
O2A	0.31610 (10)	0.78306 (19)	0.29640 (6)	0.0478 (3)	
N1A	0.49795 (10)	0.22233 (18)	0.18479 (5)	0.0281 (3)	
N2A	0.45154 (12)	0.0793 (2)	0.07075 (6)	0.0403 (4)	
C1A	0.57171 (13)	0.2789 (2)	0.23569 (7)	0.0335 (4)	
H1AA	0.6493	0.2816	0.2260	0.040*	
H1AB	0.5676	0.1805	0.2658	0.040*	
C2A	0.54431 (13)	0.4722 (2)	0.25959 (6)	0.0293 (3)	
C3A	0.63089 (12)	0.5898 (2)	0.28273 (7)	0.0314 (3)	
H3A	0.7054	0.5514	0.2798	0.038*	
C4A	0.61191 (13)	0.7628 (2)	0.31028 (7)	0.0320 (3)	
H4A	0.6714	0.8418	0.3265	0.038*	
C5A	0.50268 (13)	0.8118 (2)	0.31260 (6)	0.0303 (3)	
C6A	0.34372 (15)	0.9629 (3)	0.32388 (9)	0.0469 (5)	
H6AA	0.3181	1.0701	0.2980	0.056*	
H6AB	0.3068	0.9741	0.3592	0.056*	
C7A	0.41608 (13)	0.6991 (2)	0.28819 (7)	0.0320 (3)	
C8A	0.43308 (13)	0.5274 (2)	0.26208 (7)	0.0316 (3)	
H8A	0.3727	0.4495	0.2464	0.038*	
C9A	0.51724 (14)	0.0216 (2)	0.17120 (7)	0.0342 (4)	
H9AA	0.5076	−0.0591	0.2050	0.041*	
H9AB	0.5947	0.0056	0.1621	0.041*	
C10A	0.43710 (14)	−0.0439 (3)	0.12078 (8)	0.0403 (4)	
H10A	0.4521	−0.1794	0.1117	0.048*	
H10B	0.3596	−0.0343	0.1303	0.048*	
C11A	0.43605 (16)	0.2859 (3)	0.08385 (8)	0.0441 (4)	
H11A	0.3584	0.3089	0.0917	0.053*	
H11B	0.4505	0.3653	0.0505	0.053*	
C12A	0.51592 (14)	0.3411 (2)	0.13567 (7)	0.0356 (4)	
H12A	0.5935	0.3251	0.1268	0.043*	
H12B	0.5049	0.4777	0.1451	0.043*	
H2AA	0.522 (2)	0.062 (3)	0.0623 (9)	0.053 (6)*	
H2AB	0.403 (2)	0.044 (3)	0.0403 (10)	0.056 (6)*	

### Atomic displacement parameters (Å^2^)

**Table:**

	*U*^11^	*U*^22^	*U*^33^	*U*^12^	*U*^13^	*U*^23^
O1B	0.0238 (6)	0.0469 (7)	0.0338 (6)	−0.0022 (5)	−0.0014 (4)	0.0020 (5)
O2B	0.0567 (9)	0.0563 (9)	0.0564 (9)	−0.0110 (7)	−0.0116 (7)	−0.0159 (7)
O3B	0.0560 (9)	0.0345 (7)	0.0808 (11)	0.0035 (6)	0.0096 (7)	−0.0108 (7)
O4B	0.0263 (6)	0.0505 (8)	0.0615 (8)	0.0095 (5)	0.0023 (5)	−0.0064 (6)
O5B	0.0281 (6)	0.0474 (7)	0.0620 (8)	−0.0078 (5)	0.0014 (5)	−0.0133 (6)
O6B	0.0429 (8)	0.0581 (9)	0.0773 (10)	0.0081 (6)	0.0021 (7)	−0.0352 (8)
O7B	0.0349 (7)	0.0571 (9)	0.0762 (10)	0.0191 (6)	−0.0069 (7)	−0.0085 (7)
N1B	0.0373 (8)	0.0353 (8)	0.0397 (8)	−0.0064 (6)	0.0069 (6)	−0.0046 (6)
N2B	0.0244 (7)	0.0388 (7)	0.0360 (7)	0.0011 (5)	0.0027 (5)	0.0025 (6)
N3B	0.0278 (7)	0.0346 (7)	0.0495 (9)	0.0025 (6)	0.0055 (6)	−0.0040 (6)
C1B	0.0243 (7)	0.0346 (8)	0.0227 (7)	0.0000 (6)	0.0007 (5)	0.0048 (6)
C2B	0.0294 (8)	0.0300 (8)	0.0257 (7)	−0.0016 (6)	0.0021 (6)	0.0011 (6)
C3B	0.0276 (8)	0.0313 (7)	0.0273 (7)	0.0038 (6)	0.0051 (6)	0.0033 (6)
C4B	0.0228 (7)	0.0342 (8)	0.0259 (7)	0.0007 (6)	0.0015 (6)	0.0033 (6)
C5B	0.0275 (8)	0.0303 (7)	0.0269 (7)	−0.0010 (6)	0.0023 (6)	−0.0003 (6)
C6B	0.0258 (8)	0.0304 (8)	0.0304 (7)	0.0036 (6)	0.0035 (6)	0.0015 (6)
O1A	0.0320 (6)	0.0398 (7)	0.0528 (8)	0.0017 (5)	0.0007 (5)	−0.0153 (5)
O2A	0.0259 (6)	0.0481 (7)	0.0684 (9)	0.0020 (5)	0.0013 (6)	−0.0207 (6)
N1A	0.0256 (6)	0.0306 (6)	0.0271 (6)	−0.0010 (5)	−0.0008 (5)	−0.0014 (5)
N2A	0.0240 (7)	0.0642 (10)	0.0313 (7)	0.0038 (6)	−0.0036 (6)	−0.0126 (7)
C1A	0.0297 (8)	0.0351 (8)	0.0336 (8)	0.0028 (6)	−0.0054 (6)	−0.0029 (6)
C2A	0.0270 (8)	0.0328 (8)	0.0269 (7)	−0.0014 (6)	−0.0015 (6)	0.0004 (6)
C3A	0.0222 (7)	0.0366 (8)	0.0350 (8)	−0.0011 (6)	0.0006 (6)	0.0003 (6)
C4A	0.0251 (8)	0.0361 (8)	0.0334 (8)	−0.0062 (6)	−0.0019 (6)	−0.0013 (6)
C5A	0.0323 (8)	0.0306 (8)	0.0277 (7)	−0.0021 (6)	0.0015 (6)	−0.0017 (6)
C6A	0.0312 (9)	0.0449 (10)	0.0639 (12)	0.0027 (7)	0.0025 (8)	−0.0148 (9)
C7A	0.0228 (7)	0.0391 (8)	0.0335 (8)	−0.0006 (6)	0.0004 (6)	−0.0003 (6)
C8A	0.0244 (7)	0.0355 (8)	0.0333 (8)	−0.0043 (6)	−0.0032 (6)	−0.0040 (6)
C9A	0.0347 (8)	0.0313 (8)	0.0353 (8)	0.0012 (6)	−0.0018 (7)	−0.0021 (6)
C10A	0.0316 (9)	0.0432 (9)	0.0451 (10)	−0.0040 (7)	−0.0001 (7)	−0.0112 (8)
C11A	0.0422 (10)	0.0556 (11)	0.0332 (9)	0.0114 (8)	−0.0009 (7)	0.0044 (8)
C12A	0.0379 (9)	0.0355 (8)	0.0330 (8)	0.0006 (7)	0.0029 (7)	0.0023 (6)

### Geometric parameters (Å, º)

**Table:**

O1B—C1B	1.2597 (18)	N2A—H2AA	0.91 (2)
O2B—N1B	1.226 (2)	N2A—H2AB	0.91 (2)
O3B—N1B	1.216 (2)	C1A—H1AA	0.9900
O4B—N2B	1.2298 (18)	C1A—H1AB	0.9900
O5B—N2B	1.2234 (18)	C1A—C2A	1.511 (2)
O6B—N3B	1.2240 (19)	C2A—C3A	1.390 (2)
O7B—N3B	1.2278 (18)	C2A—C8A	1.406 (2)
N1B—C2B	1.465 (2)	C3A—H3A	0.9500
N2B—C4B	1.4505 (19)	C3A—C4A	1.400 (2)
N3B—C6B	1.456 (2)	C4A—H4A	0.9500
C1B—C2B	1.441 (2)	C4A—C5A	1.371 (2)
C1B—C6B	1.441 (2)	C5A—C7A	1.380 (2)
C2B—C3B	1.369 (2)	C6A—H6AA	0.9900
C3B—H3B	0.9500	C6A—H6AB	0.9900
C3B—C4B	1.388 (2)	C7A—C8A	1.371 (2)
C4B—C5B	1.381 (2)	C8A—H8A	0.9500
C5B—H5B	0.9500	C9A—H9AA	0.9900
C5B—C6B	1.375 (2)	C9A—H9AB	0.9900
O1A—C5A	1.3735 (19)	C9A—C10A	1.513 (2)
O1A—C6A	1.428 (2)	C10A—H10A	0.9900
O2A—C6A	1.432 (2)	C10A—H10B	0.9900
O2A—C7A	1.3757 (19)	C11A—H11A	0.9900
N1A—C1A	1.4621 (19)	C11A—H11B	0.9900
N1A—C9A	1.460 (2)	C11A—C12A	1.515 (2)
N1A—C12A	1.456 (2)	C12A—H12A	0.9900
N2A—C10A	1.481 (2)	C12A—H12B	0.9900
N2A—C11A	1.489 (2)		
			
O2B—N1B—C2B	118.49 (14)	C8A—C2A—C1A	120.69 (14)
O3B—N1B—O2B	123.68 (15)	C2A—C3A—H3A	118.9
O3B—N1B—C2B	117.80 (14)	C2A—C3A—C4A	122.20 (14)
O4B—N2B—C4B	118.01 (14)	C4A—C3A—H3A	118.9
O5B—N2B—O4B	123.24 (14)	C3A—C4A—H4A	121.9
O5B—N2B—C4B	118.76 (13)	C5A—C4A—C3A	116.24 (14)
O6B—N3B—O7B	123.10 (15)	C5A—C4A—H4A	121.9
O6B—N3B—C6B	117.76 (14)	O1A—C5A—C7A	110.17 (14)
O7B—N3B—C6B	119.13 (14)	C4A—C5A—O1A	127.81 (14)
O1B—C1B—C2B	123.00 (14)	C4A—C5A—C7A	122.01 (15)
O1B—C1B—C6B	124.81 (14)	O1A—C6A—O2A	108.21 (13)
C6B—C1B—C2B	112.14 (13)	O1A—C6A—H6AA	110.1
C1B—C2B—N1B	118.95 (13)	O1A—C6A—H6AB	110.1
C3B—C2B—N1B	116.50 (13)	O2A—C6A—H6AA	110.1
C3B—C2B—C1B	124.54 (14)	O2A—C6A—H6AB	110.1
C2B—C3B—H3B	120.7	H6AA—C6A—H6AB	108.4
C2B—C3B—C4B	118.51 (14)	O2A—C7A—C5A	109.66 (14)
C4B—C3B—H3B	120.7	C8A—C7A—O2A	127.79 (14)
C3B—C4B—N2B	118.85 (13)	C8A—C7A—C5A	122.51 (15)
C5B—C4B—N2B	119.31 (14)	C2A—C8A—H8A	121.6
C5B—C4B—C3B	121.83 (14)	C7A—C8A—C2A	116.75 (14)
C4B—C5B—H5B	120.8	C7A—C8A—H8A	121.6
C6B—C5B—C4B	118.45 (14)	N1A—C9A—H9AA	109.5
C6B—C5B—H5B	120.8	N1A—C9A—H9AB	109.5
C1B—C6B—N3B	118.73 (13)	N1A—C9A—C10A	110.87 (13)
C5B—C6B—N3B	116.76 (13)	H9AA—C9A—H9AB	108.1
C5B—C6B—C1B	124.50 (14)	C10A—C9A—H9AA	109.5
C5A—O1A—C6A	105.68 (12)	C10A—C9A—H9AB	109.5
C7A—O2A—C6A	105.78 (12)	N2A—C10A—C9A	108.90 (14)
C9A—N1A—C1A	109.85 (12)	N2A—C10A—H10A	109.9
C12A—N1A—C1A	111.28 (13)	N2A—C10A—H10B	109.9
C12A—N1A—C9A	109.25 (12)	C9A—C10A—H10A	109.9
C10A—N2A—C11A	111.56 (14)	C9A—C10A—H10B	109.9
C10A—N2A—H2AA	107.4 (14)	H10A—C10A—H10B	108.3
C10A—N2A—H2AB	110.1 (15)	N2A—C11A—H11A	109.9
C11A—N2A—H2AA	108.3 (14)	N2A—C11A—H11B	109.9
C11A—N2A—H2AB	109.7 (15)	N2A—C11A—C12A	109.15 (14)
H2AA—N2A—H2AB	110 (2)	H11A—C11A—H11B	108.3
N1A—C1A—H1AA	108.8	C12A—C11A—H11A	109.9
N1A—C1A—H1AB	108.8	C12A—C11A—H11B	109.9
N1A—C1A—C2A	113.90 (13)	N1A—C12A—C11A	110.72 (14)
H1AA—C1A—H1AB	107.7	N1A—C12A—H12A	109.5
C2A—C1A—H1AA	108.8	N1A—C12A—H12B	109.5
C2A—C1A—H1AB	108.8	C11A—C12A—H12A	109.5
C3A—C2A—C1A	118.94 (14)	C11A—C12A—H12B	109.5
C3A—C2A—C8A	120.22 (15)	H12A—C12A—H12B	108.1
			
O1B—C1B—C2B—N1B	−3.2 (2)	O2A—C7A—C8A—C2A	−179.39 (16)
O1B—C1B—C2B—C3B	177.92 (14)	N1A—C1A—C2A—C3A	−143.67 (15)
O1B—C1B—C6B—N3B	1.8 (2)	N1A—C1A—C2A—C8A	40.7 (2)
O1B—C1B—C6B—C5B	−177.91 (14)	N1A—C9A—C10A—N2A	58.56 (18)
O2B—N1B—C2B—C1B	−32.4 (2)	N2A—C11A—C12A—N1A	−57.98 (19)
O2B—N1B—C2B—C3B	146.50 (16)	C1A—N1A—C9A—C10A	176.60 (14)
O3B—N1B—C2B—C1B	149.48 (15)	C1A—N1A—C12A—C11A	−177.88 (14)
O3B—N1B—C2B—C3B	−31.6 (2)	C1A—C2A—C3A—C4A	−174.11 (15)
O4B—N2B—C4B—C3B	−1.5 (2)	C1A—C2A—C8A—C7A	175.38 (14)
O4B—N2B—C4B—C5B	177.30 (14)	C2A—C3A—C4A—C5A	−0.8 (2)
O5B—N2B—C4B—C3B	178.82 (14)	C3A—C2A—C8A—C7A	−0.2 (2)
O5B—N2B—C4B—C5B	−2.4 (2)	C3A—C4A—C5A—O1A	179.11 (15)
O6B—N3B—C6B—C1B	−150.82 (16)	C3A—C4A—C5A—C7A	−1.2 (2)
O6B—N3B—C6B—C5B	28.9 (2)	C4A—C5A—C7A—O2A	−179.39 (15)
O7B—N3B—C6B—C1B	28.4 (2)	C4A—C5A—C7A—C8A	2.7 (3)
O7B—N3B—C6B—C5B	−151.86 (16)	C5A—O1A—C6A—O2A	7.0 (2)
N1B—C2B—C3B—C4B	−178.32 (13)	C5A—C7A—C8A—C2A	−1.8 (2)
N2B—C4B—C5B—C6B	−177.33 (13)	C6A—O1A—C5A—C4A	175.15 (17)
C1B—C2B—C3B—C4B	0.6 (2)	C6A—O1A—C5A—C7A	−4.53 (19)
C2B—C1B—C6B—N3B	179.38 (13)	C6A—O2A—C7A—C5A	4.0 (2)
C2B—C1B—C6B—C5B	−0.4 (2)	C6A—O2A—C7A—C8A	−178.15 (17)
C2B—C3B—C4B—N2B	177.29 (13)	C7A—O2A—C6A—O1A	−6.8 (2)
C2B—C3B—C4B—C5B	−1.5 (2)	C8A—C2A—C3A—C4A	1.6 (2)
C3B—C4B—C5B—C6B	1.4 (2)	C9A—N1A—C1A—C2A	−169.57 (13)
C4B—C5B—C6B—N3B	179.77 (13)	C9A—N1A—C12A—C11A	60.66 (17)
C4B—C5B—C6B—C1B	−0.5 (2)	C10A—N2A—C11A—C12A	56.16 (19)
C6B—C1B—C2B—N1B	179.19 (13)	C11A—N2A—C10A—C9A	−56.31 (18)
C6B—C1B—C2B—C3B	0.3 (2)	C12A—N1A—C1A—C2A	69.32 (17)
O1A—C5A—C7A—O2A	0.31 (19)	C12A—N1A—C9A—C10A	−61.07 (17)
O1A—C5A—C7A—C8A	−177.63 (15)		

### Hydrogen-bond geometry (Å, º)

**Table:**

*D*—H···*A*	*D*—H	H···*A*	*D*···*A*	*D*—H···*A*
N2*A*—H2*AA*···O1*B*^i^	0.91 (2)	1.86 (3)	2.7409 (19)	163 (2)
N2*A*—H2*AB*···O1*B*^ii^	0.91 (2)	1.91 (2)	2.7798 (18)	159 (2)
C4*A*—H4*A*···O6*B*^iii^	0.95	2.48	3.335 (2)	150
C3*B*—H3*B*···O3*B*^iv^	0.95	2.54	3.473 (2)	166

Symmetry codes: (i) −*x*+3/2, *y*−1/2, −*z*+1/2; (ii) *x*−1/2, −*y*+1/2, *z*−1/2; (iii) *x*, *y*+1, *z*; (iv) −*x*+1, −*y*+2, −*z*+1.
